# Comparing the probability of stroke by the Framingham risk score in hypertensive Korean patients visiting private clinics and tertiary hospitals

**DOI:** 10.1186/1471-2377-10-78

**Published:** 2010-09-08

**Authors:** Cheol Ung Choi, Chang Gyu Park

**Affiliations:** 1 Cardiovascular Center, Korea University Guro Hospital, Seoul, Korea

## Abstract

**Background:**

The purpose of this study was to investigate the pattern of distribution of risk factors for stroke and the 10-year probability of stroke by the Framingham risk score in hypertensive patients visiting private clinics vs. tertiary hospitals.

**Methods:**

A total of 2,490 hypertensive patients who attended 61 private clinics (1088 patients) and 37 tertiary hospitals (1402 patients) were enrolled. The risk factors for stroke were evaluated using a series of laboratory tests and physical examinations, and the 10-year probability of stroke was determined by applying the Framingham stroke risk equation.

**Results:**

The proportion of patients who had uncontrolled hypertension despite the use of antihypertensive agents was 49% (66 and 36% of patients cared for at private clinics and tertiary hospitals, respectively; p < 0.001). The average 10-year probability of stroke by the Framingham risk score in hypertensive patients was 21% (approximately 2.2 times higher than of the risk of stroke in the Korean Cancer Prevention Study [KCPS] cohort) and was higher in patients attending tertiary hospitals compared to private clinics (16 and 24% of patients attending private clinics and tertiary hospitals, respectively; p < 0.001).

**Conclusions:**

Since the 10-year probability of stroke by the Framingham risk score in hypertensive patients attending tertiary hospitals was higher than the risk for patients attending private clinics. We suggest that the more aggressive interventions are needed to prevent and early detect an attack of stroke in hypertensive patients attending tertiary hospitals.

## Background

Stroke is becoming the major cause of death and behavioral disorders because of the rapid increase of the elderly population with the elongation of life expectancy[1].

Hypertension and aging are known to be the most common risk factors for stroke worldwide[2,3]. Aging is an irreversible factor, but hypertension is controllable. It has been reported that appropriately treated hypertension reduces the risk of stroke by 40% and every 20/10 mmHg incremental increase in systolic blood pressure (SBP) and diastolic blood pressure (DBP) above115/75 mmHg doubles the risk of stroke[4]. The Framingham study in the USA, a well-regarded prospective epidemiologic study on chronic diseases initiated in 1948, elucidated that the risk factors for stroke include age, SBP, use of antihypertensive agents, diabetes, smoking, history of cardiovascular diseases (CVD), atrial fibrillation, and left ventricular hypertrophy (LVH)[5-7]. In Korea, it was reported that the average 10-year probability of stroke in the Korean Cancer Prevention Study (KCPS) cohort was 3.5% for males and 3.7% for females[8]. In addition, the average 10-year probability of stroke in the 55-84 year old group within the KCPS cohort was 10% for males and 9% for females[8]. In Western countries, the 10-year risk of stroke in hypertensive patients is approximately 3-4 times higher than the risk of stroke in the general population[9]. We reported that the 10-year probability of stroke by the Framingham risk score in hypertensive Korean patients was approximately 1.7 times higher than of the risk of stroke in of the KCPS cohort[10]. However, this study population was derived from a highly selected group of patients who were attending private clinics and the results did not reflect the real 10-year probability of stroke in Korean hypertensive patients. Therefore, we set out to determine the 10-year probability of stroke in hypertensive patients attending tertiary hospitals in addition to private clinics using the Framingham risk score and we planned to compare the 10-year probability of stroke by the Framingham risk score between the two groups.

## Methods

### Study population

We conducted a multicenter study evaluating patients with hypertension, who attended 61 private clinics and 37 tertiary hospitals between 2004 and 2006 in Korea. Inclusion criteria were (1) 55-84 years of age; (2) subjects who had attended constantly the same private clinics or tertiary hospitals for at least 2 years. Exclusion criteria were (1) renal insufficiency (Cr > 2.0 mg/dl); (2) malignancy; (3) subjects who had history of stroke, including transient ischemic attacks; (4) subjects who offered overlapped service by primary and tertiary centers during the study. Consequently, 2490 patients (1088 patients in 61 private clinics and 1402 patients in tertiary hospitals) were enrolled for the study. Among those 2490 patients, 1648 patients (940 patients in 61 private clinics and 708 patients in tertiary hospitals) have not been hospitalized before. The other 842 patients have been hospitalized before, but they have not been hospitalized during the 2 years study periods. Therefore all patients were selected from outpatient clinics in the both private clinics and tertiary hospitals. All subjects gave written informed consent. This study was approved by the local ethics committee.

### Laboratory and lifestyle factors measurement

The clinical evaluation, laboratory measurements and blood pressure measurements were performed at outpatient clinics at all through the morning. Measurement of blood pressure (BP) was performed with a mercury sphygmomanometer by trained technicians according to a standardized protocol[11]. The BP was measured in the right arm, using an appropriately-sized cuff and a standard mercury sphygmomanometer, after the subjects had been seated for at least 15 min, with the subject's feet on the floor and the arm supported at the level of the heart. We used phase V Korotkoff sounds as the diastolic point, as specified by the guideline[11]. The BP measurements for the participants were repeated twice after a 30-s interval, and were recorded to the nearest 2 mmHg. The average value of the readings was used as a measure of the SBP and DBP. Fasting blood samples were obtained on the morning after at least 8 hours of fasting. Plasma glucose, total cholesterol, triglycerides, high density lipoprotein (HDL)-cholesterol, and low density lipoprotein (LDL)-cholesterol were measured. The risk factors of stroke included age, diabetes, smoking, a history of CVD, atrial fibrillation, LVH, dyslipidemia, obesity, and a family history of stroke. The questionnaire required subjects to estimate the smoking history and the family history of stroke.

### Estimation of 10-year probability of stroke

The 10-year probability of stroke was estimated using the Framingham risk score[5]. The variables used in the Framingham risk score are age, systolic blood pressure, antihypertensive therapy, diabetes mellitus, smoking, history of CVD, atrial fibrillation, and LVH.

### Study definitions

Hypertension was defined as repeated measurements of ≥ 140 mmHg (SBP) or ≥ 90 mmHg (DBP) or a previous diagnosis of hypertension. The stage of hypertension was classified according to the Joint National Committee 7 (JNC-7) criteria [11]: normal (SBP < 120 mm Hg and DBP < 80 mm Hg); pre- hypertension (120 < SBP < 140 mm Hg or 80 < DBP < 90 mm Hg); stage 1 (140 < SBP < 160 mm Hg or 90 < DBP < 100 mm Hg); and stage 2 (SBP > 160 or DBP ≥ 100 mm Hg). Uncontrolled hypertension was defined as repeated measurements of ≥ 140 mmHg SBP and ≥ 90 mmHg DBP, despite the use of antihypertensive agents. Diabetes was defined as fasting blood glucose concentration ≥ 126 mg/dl or taking diabetes medications. Subjects were classified into two groups with respect to smoking: non-smoker and current-smoker. Atrial fibrillation was confirmed by electrocardiogram. The product of the duration of the QRS interval times the Cornell voltage combination (R_aVL _+ S_V3_, with 6 mm added for females; > 2440 mm · msec [12,13]) or Sokolow-Lyon voltage (S_V1 _+ RV_5/6_; > 38 mm [14]) was used to identify LVH.

The history of CVD was defined as follows: history of coronary artery disease (CAD; defined as any hospitalization for acute myocardial infarction or angina) and history of congestive heart failure (CHF; defined as any hospitalization for CHF). If stroke had developed in a first degree relative, subjects were considered to have a family history of stroke. Dyslipidemia was defined as follows: patients receiving lipid-lowering agents after the diagnosis of dyslipidemia or patients in whom the total cholesterol was > 240 mg/dL. A tertiary hospitals were defined as teaching hospitals with 500-1500 bed facilities or major hospital that usually has a full complement of services including pediatrics, general medicine, various branches of surgery and psychiatry or a specialty hospital dedicated to specific sub-specialty care. Private clinics were defined as those with less than 100 bed facilities and without full specific sub-specialty care. Allocation of individual patients to private clinics or tertiary hospitals was decided by patients' discretion, because Korean patients can any time attend tertiary hospitals without private clinics' referral.

### Statistical analysis

Statistical analysis was performed using the SPSS 10.0 software package (SPSS, Inc., Chicago, IL, USA). Continuous variables were expressed as the means (SD) and categorical variables were reported as the number (%). Continuous variables were compared using the Student t-test or one way-ANOVA. When we used one-way ANOVA, *post hoc *multiple comparisons were performed using Duncan (D) multiple comparison tests. Non-parametric analysis for one way-ANOVA was performed using Kruscal-Wallis analysis. Categorical variables were compared using chi-square or Fisher's exact tests. To evaluate the factors associated with uncontrolled hypertension, multivariate logistic regression analysis was performed and to evaluate correlation coefficient between various factors and 10-year risk of stroke, multivariate regression analysis was performed. A p < 0.05 was considered statistically significant.

## Results

### Baseline characteristics and risk factors

The baseline characteristics and risk factors of our subjects are summarized in Table 1. The patients were comprised of 1082 males and 1408 females. The mean age of patients was 68.1 (SD 6.45) years. The average SBP was 142 (SD 20) mmHg and the average DBP was 85 (SD 13) mmHg. The tertiary hospital group was older than the private clinic group (respectively, 68.4 versus 67.7, p <0.001). In the private clinic group, SBP, DBP, diabetes, and dyslipidemia were higher and/or more frequent compared to the tertiary hospital group. In contrast, in the tertiary hospital group, the prevalence of smoking, atrial fibrillation, history of CVD, LVH, family history of stroke, and the number of patients taking lipid-lowering agents was higher compared to the private clinic group. The prevalence of subjects taking antihypertensive agents was 79% (83% of private clinic patients and 76% of tertiary hospital patients; p < 0.001; Table 1). The proportion of patients who had uncontrolled hypertension despite the use of antihypertensive agents was 49% (66% of private clinic patients and 36% of tertiary hospital patients; p < 0.001; Table 1).

**Table 1:** Characteristics of the risk factors in the study subjects

Risk factors	Private Clinics	Tertiary hospitals	Total	
		
	(n = 1088)	(n = 1402)	(n = 2490)	p
Age, years	67.7 ± 4.95	68.4 ± 7.40	68.1 ± 6.45	< 0.001
Male, n (%)	449 (41)	633 (45)	1082 (44)	0.053
Systolic blood pressure, mmHg	154 ± 17	132 ± 16	142 ± 20	< 0.001
Diastolic blood pressure, mmHg	92 ± 12	79 ± 10	85 ± 13	< 0.001
Diabetes mellitus, n (%)	234 (22)	225 (16)	459 (18)	< 0.001
Cigarette smoking, n (%)	124 (11)	429 (31)	553 (22)	< 0.001
History of CVD, n (%)	148 (14)	694 (50)	842 (34)	< 0.001
Atrial fibrillation, n (%)	43 (4)	108 (8)	151 (6)	< 0.001
Left ventricular hypertrophy, n (%)	167 (15)	452 (32)	618 (25)	< 0.001
Dyslipidemia, n (%)	185 (17)	163 (13)	348 (15)	0.002
Stroke family history, n (%)	114 (11)	189 (14)	303 (12)	0.023
Lipid-lowering therapy, n (%)	260 (24)	441 (32)	701 (28)	< 0.001
Antihypertensive agents, n (%)	903 (83)	1065 (76)	1968 (79)	< 0.001
Uncontrolled HTN, n (%)	596 (66)	384 (36)	980 (49)	< 0.001

CVD, cardiovascular disease; HTN, hypertension; Values, Mean ± ± standard deviation or number (%); n, number

### 10-year probability of stroke by the Framingham risk score

**Figure 1:**
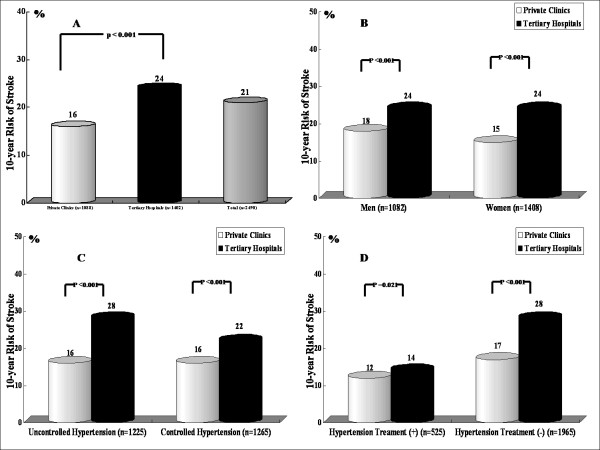
**The average 10-year risk of stroke by the Framingham risk score between patients attending private clinics and tertiary hospitals A: in all patients, B: according to gender, C: in patients with uncontrolled hypertension and controlled hypertension, D: in patients taken antihypertensive agents and not taken antihypertensive agents**. The average 10-year probability of stroke by the Framingham risk score in the tertiary hospital patients was higher than the private clinic patients independent of gender, control of hypertension and taking antihypertensive agents.

The average 10-year probability of stroke by the Framingham risk score was 21% (16% for private clinic patients and 24% of tertiary hospital patients; Figure 1-A). In both males and females, the average 10-year probability of stroke by the Framingham risk score in the tertiary hospital patients was higher than the private clinic patients (Figure 1-B). The average 10-year probability of stroke by the Framingham risk score in the tertiary hospital patients was also higher than the private clinic patients, independent of the control of hypertension and treatment of hypertension (Figure 1-C, D). As expected, the 10-year probability of stroke by the Framingham risk score in patients with hypertension gradually increased in proportion to age independent of the control of hypertension and treatment of hypertension (Table 2). The average 10-year probability of stroke by the Framingham risk score in males was higher than females (22 versus 20%).

**Table 2:** Average 10-year probability of stroke by the Framingham risk score according to age

	All patients				Patients with uncontrolled hypertension				Patients with controlled hypertension			
	**< 65** **(n = 797)**	**65-69** **(n = 735)**	**70-74** **(n = 523)**	**> 74** **(n = 435)**	**< 65** **(n = 416)**	**65-69** **(n = 382)**	**70-74** **(n = 253)**	**> 74** **(n = 174)**	**< 65** **(n = 381)**	**65-69** **(n = 353)**	**70-74** **(n = 270)**	**> 74** **(n = 261)**

Female*	12.4 ± 10.6^α^	14.8 ± 11.6^β^	22.3 ± 16.7^χ^	36.5 ± 2.3^δ^	12.5 ± 9.5^α^	16.5 ± 12.0^β^	22.3 ± 15.1^χ^	35.2 ± 21.3^δ^	10.9 ± 9.5^α^	14.4 ± 11.9^β^	21.1 ± 15.6^χ^	34.6 ± 21.2^δ^
Male*	15.7 ± 10.0^α^	20.2 ± 12.0^α^	24.6 ± 14.5^β^	34.9 ± 20.1^χ^	16.6 ± 10.4^α^	20.7 ± 12.4^β^	25.5 ± 14.8^χ^	32.9 ± 17.3^δ^	14.5 ± 9.3^α^	19.7 ± 11.6^β^	23.9 ± 14.4^χ^	36.0 ± 21.5^δ^
Total*	13.9 ± 10.4^α^	17.2 ± 12.1^β^	23.3 ± 15.9^χ^	35.9 ± 21.5^δ^	15.3 ± 11.1^α^	17.7 ± 12.1^β^	24.3 ± 16.6^χ^	36.9 ± 21.7^δ^	12.5 ± 9.5^α^	16.5 ± 12.0^β^	22.3 ± 15.1^χ^	35.2 ± 21.3^δ^

Values were presented by Mean ± standard deviation

*, P values are less than 0.001 for each entry and P-value is for one-way ANOVA analysis of variance.

^a, b, c, d ^same letters indicate statistical non-significance based on Duncan's multiple comparisons

### Influencing factors on uncontrolled hypertension

To evaluate the factors associated with uncontrolled hypertension, multivariate logistic regression analysis was performed after adjusting for age, sex, treatment of hypertension, diabetes, history of CVD, atrial fibrillation, LVH, family history of CVA, dyslipidemia, smoking and weight. Age and diabetes were associated with uncontrolled hypertension (Table 3).

**Table 3:** Influencing factors on uncontrolled hypertension using multivariate logistic regression analysis

	OR	95% CI	p
Age	0.96	0.94-0.98	0.001
Male	0.71	0.49-1.02	0.064
Treatment of hypertension	0.95	0.68-1.32	0.746
Diabetes	0.52	0.37-0.74	< 0.001
History of CVD	1.14	0.85-1.56	0.381
Atrial fibrillation	1.03	0.57-1.86	0.925
LVH	1.17	0.87-1.5	0.297
Family history of CVA	0.77	0.53-1.11	0.154
Dyslipidemia	0.87	0.59-1.28	0.479
Smoking	0.87	0.60-1.27	0.480
Weight	1.02	1.00-1.03	0.054

OR, odds ratio; CVD, cardiovascular disease; LVH, left ventricular hypertrophy; CVA, cerebrovascular accident. Multivariate analysis was performed after adjusting for age, sex, treatment of hypertension, diabetes, history of CVD, atrial fibrillation, LVH, family history of CVA, dyslipidemia, smoking and weight.

### Influencing factors on 10-year probability of stroke by the Framingham risk score

To evaluate correlation coefficient between various factors and 10-year probability of stroke by the Framingham risk score, multivariate regression analysis was performed after adjusting for age, diabetes, smoking, history of CVD, atrial fibrillation, LVH and SBP. There were relatively high correlation coefficient in atrial fibrillation and LVH (Table 4).

**Table 4:** Influencing factors on 10-year risk of stroke by the Framingham risk score using multivariate regression analysis

	ß	**R**^**2**^
Age*	1.04	0.783
Diabetes*	8.40	
Smoking*	6.90	
History of CVD*	9.28	
Atrial fibrillation*	20.29	
LVH*	17.96	
SBP*	0.18	

CVD, cardiovascular disease; LVH, left ventricular hypertrophy; SBP, systolic blood pressure. Multivariate analysis was performed after adjusting for age, diabetes, smoking, history of CVD, atrial fibrillation, LVH and SBP.*, p values are less than 0.001 for each entry.

## Discussion

This study is a report designed to evaluate the 10-year probability of stroke in hypertensive Korean patients and to compare the probability in hypertensive patients attending tertiary hospitals and private clinics using the Framingham risk score.

The main finding of the present study was that the 10-year probability of stroke by the Framingham risk score in hypertensive Korean patients was approximately 2.2 times higher than that of stroke in the KCPS cohort and the 10-year probability of stroke by the Framingham risk score in hypertensive patients attending tertiary hospitals was higher than hypertensive patients attending private clinics.

Table 1 showed the different profiles between the two groups. These different profiles could be caused by the fact that patients attending tertiary hospitals usually more severe and had more comorbidity than patients attending private clinics. Patients attending tertiary hospitals were older and had more atrial fibrillation, LVH, history of CVD, and smoking than patients attending private clinics (Table 1).

In the present study, the 10-year probability of stroke by the Framingham risk score in hypertensive patients attending the tertiary hospitals was higher than the risk in patients attending the private clinics. This result was attributed to the increased values and/or prevalence of the risk factors (age, smoking, history of CVD, atrial fibrillation, and LVH) in the tertiary hospital group than the private clinic group; there were more high risk patients in the tertiary hospital group. In addition, the correlation coefficient between various factors and 10-year probability of stroke by the Framingham risk score were higher in atrial fibrillation, LVH (Table 4). Therefore, we thought that these differences could also cause the difference in 10-year probability of stroke by the Framingham risk score between patients attending tertiary hospitals and patients attending private clinics. In other words, the higher probability of stroke in hypertensive patients attending tertiary hospitals is not entirely due to their hypertension but is due to the other factors used in the Framingham score. In Korea, most of patients (especially patients with more comorbidity) prefer the tertiary hospitals to private clinics and they can any time attend tertiary hospitals without referral of private clinics. The disposition to go tertiary hospitals could cause the different comorbidity between tertiary hospitals and private clinics and then cause a greater stroke risk in patients attending tertiary hospitals than patients attending private clinics.

In our study, the proportion of treated hypertensive patients among hypertensive Korean patients was 79% (Table 1) and the proportion of patients who had uncontrolled hypertension among patients treated with antihypertensive agents was 49% (Table 1). These results were high compared to those of the Korean National Health and Nutrition Examination Survey (2005)[15]. Since the study population was derived from a select group of patients who were suspected to have, or had established cardiac disease, the proportion of antihypertensive agents and the proportion of patients who had uncontrolled hypertension was thought to be high. In addition, age and diabetes were associated with uncontrolled hypertension (Table 3). Age cannot be controlled but, diabetes can be controllable. Therefore, these results suggest that appropriate management of diabetes could be an important factor in the control of hypertension.

In Western countries, the 10-year risk of stroke in hypertensive patients was approximately from 3-4 times higher than stroke in the general population[9]. We reported that probability of stroke in Korean hypertensive patients attending community based-hospitals[10]. This study is the first study to compare the 10-year probability of stroke in hypertensive Korean patients attending tertiary hospitals and private clinics based on the Framingham risk score. As mentioned (*vide supra)*, hypertension and aging are known to be most common risk factors of stroke and hypertension is controllable. In Korea, since the proportion of patients who have uncontrolled hypertension despite use of antihypertensive agents was 49% (Table 1), we suggest that the appropriate control of BP is an important approach to prevent the risk of stroke. In addition, the control of risk factors, such as SBP, antihypertensive therapy, diabetes mellitus, smoking, history of CVD, atrial fibrillation, and LVH, was thought to also be important for the preventing stroke since these risk factors were common in hypertensive patients.

This study has some limitations. First, the study population is not representative of a real hypertension population in Korea. Because it was restricted to patients in the population between 55 and 84 years of age, and did not include patients attending the other public health centers. Although it would be appropriate to include private clinics, tertiary hospitals, and other public health centers in the study, our study did not include hypertensive patients from other public health centers. Therefore, the cardiovascular risk factors were overrepresented and it cannot be denied that the enrolled subjects were the hypertensive patients with a higher risk than the actual hypertension population.

Second, since patients may see the tertiary center before the primary and secondary levels, it could be possible that there was an overlap in services offered by primary and tertiary centers. Since we had the information about a resident registration number of all patients, we confirmed that there was not overlap in services offered by primary and tertiary centers during progression of the study. In addition, we enrolled subjects who had attended constantly the same private clinics or tertiary hospitals for at least 2 years, therefore there was not overlapped in services offered by primary and tertiary centers for at least 2 years

Third, we predicted the 10-year probability of stroke in hypertensive patients using the Framingham risk score. However, Framingham risk score by hypertensive status when these people must have higher scores because of their hypertension. In addition, these people have higher Framingham risk score because hypertensive patients had more comorbidity than general population. Therefore, 10-year probability of stroke in our subjects could be overestimated.

Fourth, the variables used in the Framingham risk score are associated with the duration of hypertension. Therefore, the duration of hypertension could be an important factor and have an effect on Framingham risk score. However, we had no data about the duration of hypertension.

Fifth, this study was a simple cross-section study and we had no information about the practical stroke event. Therefore, we did not compare the 10-year probability of stroke by the Framingham risk score with the actual stroke incidence. In addition, risk factors were uneven distributed between the two groups. Some risk factors (old age, smoking, afib, LVH, history of CVD etc.) were more in tertiary hospitals, and other risk factors (high BP, frequency of diabetes and dyslipidemia) were more in private clinics. Therefore, whether real strokes will be more or less frequent over 10 years in those treated at larger institutions remains unknown. If a well-controlled, large prospective study is conducted for hypertensive patients, we could analyze the relationship between the 10-year probability of stroke by the Framingham risk score and the actual stroke incidence.

Sixth, the Framingham stroke score in Korean population have not been validated yet. The Framingham Heart Study has contributed to the identification of risk factors for stroke and has developed multivariate functions to predict absolute stroke risk. However, there have been no large cohorts that compare the practical stroke event and probability of stroke. In addition, other ethnic populations may differ from Caucasians or people living in suburban as Framingham in terms of diet, life style, social environment, or genetic predisposition. Therefore, in Korean population, there are limitations in applications of the risk functions obtained from the Framingham study.

Seventh, we measured the smoking history and the family history of stroke with a single, self-reported questionnaire. Therefore, a non-differential misclassification was possible.

## Conclusion

The average 10-year probability of stroke by the Framingham risk score in hypertensive Korean patients was 21% and the 10-year risk of stroke by the Framingham risk score in hypertensive patients attending tertiary hospitals (24%) was higher than the patients attending private clinics (16%). Therefore, we suggest that the more aggressive interventions are needed to prevent and early detect an attack of stroke in hypertensive patients especially attending tertiary hospitals.

## Competing interests

The authors declare that they have no competing interests.

## Authors' contributions

CUC participated in the design of the study, analyzed and interpreted data and drafted the manuscript. CGP conceived of the study and participated in its design and critically revised the manuscript. All authors read and approved the final manuscript.

## Pre-publication history

The pre-publication history for this paper can be accessed here:

http://www.biomedcentral.com/1471-2377/10/78/prepub
